# Thioredoxin overexpression in mitochondria showed minimum effects on aging and age-related diseases in male C57BL/6 mice

**DOI:** 10.31491/apt.2020.03.009

**Published:** 2020-03-27

**Authors:** Madeline G. Roman, Lisa C. Flores, Geneva M. Cunningham, Christie Cheng, Sara Dube, Colton Allen, Holly Van Remmen, Yidong Bai, Gene B. Hubbard, Thomas L. Saunders, Yuji Ikeno

**Affiliations:** a Barshop Institute for Longevity and Aging Studies, The University of Texas Health Science Center at San Antonio, San Antonio, TX, USA; b Aging and Metabolism Research Program, Oklahoma Medical Research Foundation, Oklahoma City, OK, USA; c Department of Cell Systems and Anatomy, The University of Texas Health Science Center at San Antonio, San Antonio, TX, USA; d Department of Pathology, The University of Texas Health Science Center at San Antonio, San Antonio, TX, USA; e Transgenic Animal Model Core, University of Michigan, Ann Arbor, MI, USA; f Geriatric Research Education and Clinical Center (GRECC), Audie L. Murphy VA Hospital, South Texas Veterans Health Care System, San Antonio, TX, USA

**Keywords:** Thioredoxin, transgenic Mouse, oxidative stress, aging

## Abstract

**Objective:**

In this study, the effects of overexpression of thioredoxin 2 (Trx2) on aging and age-related diseases were examined using Trx2 transgenic mice [Tg(TXN2]^+/0^]. Because our previous studies demonstrated that thioredoxin (Trx) overexpression in the cytosol (Trx1) did not extend maximum lifespan, this study was conducted to test if increased Trx2 expression in mitochondria shows beneficial effects on aging and age-related pathology.

**Methods:**

Trx2 transgenic mice were generated using a fragment of the human genome containing the TXN2 gene. Effects of Trx2 overexpression on survival, age-related pathology, oxidative stress, and redox-sensitive signaling pathways were examined in male Tg(TXN2)^+/0^ mice.

**Results:**

Trx2 levels were significantly higher (approximately 1.6- to 5-fold) in all of the tissues we examined in Tg(TXN2)^+/0^ mice compared to wild-type (WT) littermates, and the expression levels were maintained during aging (up to 22-24 months old). Trx2 overexpression did not alter the levels of Trx1, glutaredoxin, glutathione, or other major antioxidant enzymes. Overexpression of Trx2 was associated with reduced reactive oxygen species (ROS) production from mitochondria and lower isoprostane levels compared to WT mice. When we conducted the survival study, male Tg(TXN2)^+/0^ mice showed a slight extension (approximately 8-9%] of mean, median, and 10th percentile lifespans; however, the survival curve was not significantly different from WT mice. Cross-sectional pathological analysis (22-24 months old) showed that Tg(TXN2)^+/0^ mice had a slightly higher severity of lymphoma; however, tumor burden, disease burden, and severity of glomerulonephritis and inflammation were similar to WT mice. Trx2 overexpression was also associated with higher c-Jun and c-Fos levels; however, mTOR activity and levels of NFκB p65 and p50 were similar to WT littermates.

**Conclusions:**

Our findings suggest that the increased levels of Trx2 in mitochondria over the lifespan in Tg(TXN2)^+/0^ mice showed a slight life-extending effect, reduced ROS production from mitochondria and oxidative damage to lipids, but showed no significant effects on aging and age-related diseases.

## Introduction

Thioredoxin (Trx) has drawn much interest in biology, including aging, because of its unique ability to attenuate the level of oxidative stress/damage and alter redox-sensitive signaling, which could have diverse effects on pathophysiology. Trx is a small protein (12kDa) with two redox-active cysteine residues in the active center (Cys-Gly-Pro-Cys) [1] and acts as the reductant for a variety of enzymes [1–7]. Trx also plays an important role in maintaining a reduced environment in cells through thiol-disulfide exchange reactions [1]. This rapid and readily reversible reaction is ideal for protein function control via the redox state of structural or catalytic SH groups. In humans, Trx1 and Trx2 have been identified in different compartments of the cell, i.e., Trx1 in cytosol [8] and Trx2 in mitochondria [9].

To test the pathophysiological roles of Trx1 during aging, two transgenic mice overexpressing Trx1 were generated using: 1) a transgene containing the human thioredoxin cDNA fused to the β–actin promoter [Tg(act-TXN)^+/0^ mice] [10] and 2) clones of the human TXN gene containing endogenous promoters [Tg(TXN)^+/0^] [11]. The initial aging study demonstrated that Tg(act-TXN)^+/0^ mice had an increased lifespan compared to their WT littermates [10,12]. However, the lifespan of WT C57BL/6 mice in their colony was shorter than WT C57BL/6 mice in aging colonies under optimal conditions, which indicates that the study was conducted under unconventional housing conditions. Therefore, an aging study with the same line of Tg(act-TXN)^+/0^ mice under optimal housing conditions was conducted by our laboratory to examine the effects of increased levels of Trx1 on aging and age-related diseases. Under optimal housing conditions, male and female Tg(act-TXN)^+/0^ mice showed an extension of lifespan only in the earlier part of their lifespan; however, no increase in maximum lifespan was observed [13]. Because Tg(act-TXN)^+/0^ mice showed that the levels of overexpression significantly decreased with age possibly due to the β-actin promoter, we subsequently generated new transgenic mice with clones of the human TXN gene containing endogenous promoters [Tg(TXN)^+/0^] to ensure that the transgene would be overexpressed throughout the lifespan [11]. The aging study with Tg(TXN)^+/0^ mice demonstrated that continuous overexpression of Trx1 over the lifespan slightly extended the earlier part of life, but had no significant effects on median or maximum lifespans. Tg(TXN)^+/0^ mice also showed that Trx1 overexpression accelerates cancer development in old mice [11], which is similar to the pathology results in Tg(act-TXN)^+/0^ mice [13]. The results from two lines of Trx1 transgenic mice showed that overexpression of Trx1 in cytosol does not have beneficial effects in aged mice, i.e., no life-extending effects with enhanced tumor development in the later part of life.

These results led us to question whether increased levels of Trx2 in mitochondria could play more important roles in aging, i.e., extend both the earlier and later part of lifespan and attenuate the development of age-related diseases. The study with mCAT mice [14] clearly demonstrated the importance of antioxidant overexpression in mitochondria in aging. In their study, overexpressing catalase in mitochondria significantly increased lifespan and reduced some types of cancers, while overexpressing catalase in other compartments of the cells (nucleus or peroxisome) did not change lifespan [14].

Thus, the purpose of this study is to examine the effects of Trx2 overexpression in the mitochondria on aging. We conducted a survival study using transgenic mice generated with a clone of the human TXN2 gene containing an endogenous promoter [Tg(TXN2)^+/0^]. Here, we report that increased levels of Trx in mitochondria [Tg(TXN2)^+/0^ mice] showed no significant changes in lifespan compared to WT mice, although a slight extension (approximately 8-9%) of mean, median, and 10th percentile lifespans (not statistically significant) was observed. Tg(TXN2)^+/0^ mice also showed no significant effects on age-related pathological changes. Therefore, our results suggest that the overexpression of Trx2 has minimal effects on aging and development of age-related diseases in male C57BL/6 mice.

## Materials and methods

### Animals and animal husbandry

**Figure 1.**
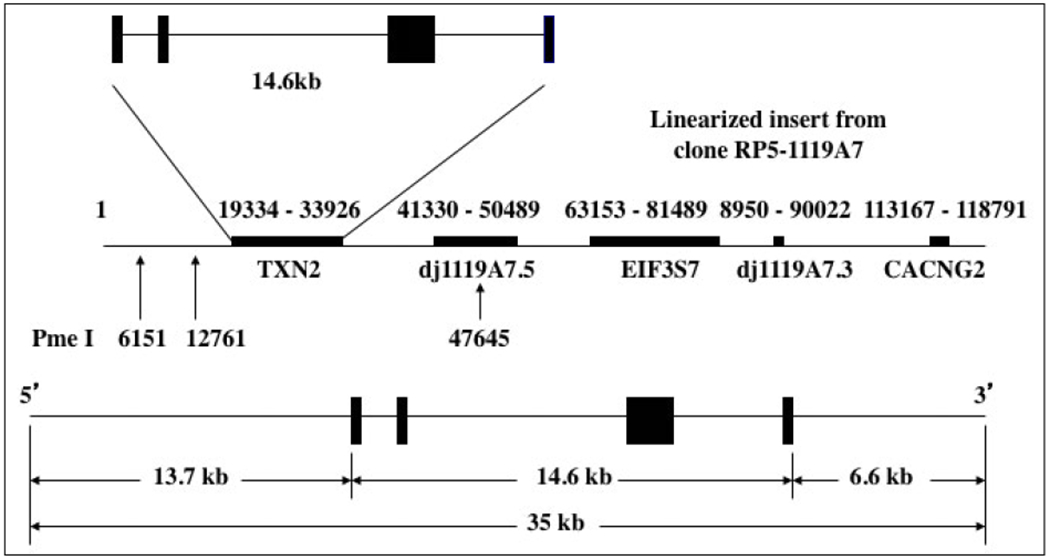
DNA construct for Tg(TXN2)^+/0^ mice. The human thioredoxin 2 gene (TXN2) with 13.7 kb and 6.6 kb of the 5’- and 3’-flanking sequences was used to generate Tg(TXN2)^+/0^ mice by pronuclear microinjection of zygotes from the mating of (C57BL/6J X SJL/J)F1 females with (C57BL/6J X SJL/J)F1 males.

We generated the Tg(TXN2)^+/0^ mice using the human thioredoxin 2 gene [a PAC clone (RP5-1119A7), Children’s Hospital Oakland Research Institute’s (CHORI) BACPAC Resources Center (BPRC), Oakland, CA], which contained the TXN2 gene and 13.7 kb and 6.6 kb of the 5’- and 3’-flanking sequences, respectively (Figure 1). These transgenic mice were produced by pronuclear microinjection of zygotes obtained from the mating of (C57BL/6J X SJL/J)F1 females with (C57BL/6J X SJL/J) F1 males (Jackson Laboratory, stock no. 100012) and were backcrossed to C57BL/6 mice ten times. Male hemizygous transgenic mice [Tg(TXN2)^+/0^] were crossed with C57BL/6 females to generate hemizygous transgenic and WT control mice. All mice were fed a commercial chow (Teklad Diet LM485: Madison, WI) and acidified (pH=2.6-2.7) filtered reverse osmosis water ad libitum. To measure the amount of food consumption, the amount of chow removed from the cage hopper and the spillage (the chow on the bottom of the cage) were weighed monthly. Actual food consumed was calculated by subtracting the spillage from the chow removed from the hopper. All of the mice were weighed monthly. The mice were maintained pathogen-free in microisolator units on Tek FRESH ultra laboratory bedding. Sentinel mice housed in the same room and exposed weekly to bedding collected from the cages of experimental mice were sacrificed on receipt and every six months thereafter for monitoring of viral antibodies (Mouse Level II Complete Antibody Profile CARB, Ectro, EDIM, GDVII, LCM, M. Ad-FL, M. Ad-K87, MCMV, MHV, M. pul., MPV, MVM, Polyoma, PVM, Reo, Sendai; BioReliance, Rockville, MD). All tests were negative.

We chose young (4-6 months old) and old (22-24 months old) age groups for the experiments described below because: 1) C57BL/6 mice reach their optimum reproductivity at 3-7 months of age and 2) have increased incidence of cancers after 20 months of age followed by advanced stages of cancer around 26-30 months of age. For some of the assays, only liver was used because there is a large amount of information available with respect to age-related changes, and liver is one of the major sites for the development of spontaneous tumors in C57BL/6 mice.

### Determination of Trx2 expression

Thioredoxin 2 (Trx2) levels were measured using the mitochondrial fraction obtained from several tissues (liver, kidney, heart, brain, lung, spleen, and testes) from young (4-6 months old) and liver from old (22-24 months old) Tg(TXN2)^+/0^ and WT mice as previously described [13,15]. Western blot analysis was performed using rabbit anti-Trx2 polyclonal antibody (Catalog No. LF-PA0012; LabFrontier, Seoul, South Korea). After incubation with the primary antibodies, membranes were incubated with the respective peroxidase-linked secondary antibodies (Catalog No. P0217; Dako, Carpinteria, CA). Chemiluminescence was detected using the ECL Western blot detection kit (Amersham Biosciences Corp., Piscataway, NJ).

### Trx1 levels

Cytosolic fractions obtained from tissues homogenized as previously described [13] were used to determine Trx1 levels in liver obtained from young (4-6 months old) Tg(TXN2)^+/0^ and WT mice by Western blot analysis using goat anti-human Trx1 polyclonal antibodies (Catalog No. 705; American Diagnostica, Inc., Greenwich, CT). These antibodies recognize total Trx1 (both oxidized and reduced forms). After incubation with the primary antibody, membranes were incubated with the peroxidase-linked secondary antibody (Catalog No. P0449; Dako, Carpinteria, CA). Chemiluminescence was detected with an ECL Western blot detection kit (Amersham Biosciences Corp., Piscataway, NJ).

### Glutaredoxin and total glutathione levels

Glutaredoxin (Grx) levels were measured using total homogenate fractions obtained from the liver of young (4-6 months old) Tg(TXN2)^+/0^ and WT mice as previously described [13]. Western blot analysis was performed using goat anti-human glutaredoxin polyclonal antibody (Catalog No. 710; American Diagnostica, Inc., Greenwich, CT). After incubation with the primary antibodies, membranes were incubated with the respective peroxidase-linked secondary antibodies (Catalog No. P0449; Dako, Carpinteria, CA). Chemiluminescence was detected using the ECL Western blot detection kit (Amersham Biosciences Corp., Piscataway, NJ). The levels of total glutathione were determined using the Bioxytech GSH-420 kit (Catalog No. 21023; Oxis International, Inc., Foster City, CA) in several tissues (liver, kidney, heart, brain, lung, spleen, and testes) from young (4-6 months old) Tg(TXN2)^+/0^ and WT mice.

### Determination of major antioxidant enzyme activities: Cu/ZnSOD, MnSOD, glutathione peroxidase, and catalase

The activities of major antioxidant enzymes (Cu/ZnSOD, MnSOD, glutathione peroxidase (GPx), and catalase) were measured in tissue homogenates obtained from the liver of young (4-6 months old) Tg(TXN2)^+/0^ and WT mice. The supernatants were used for the antioxidant defense enzymatic activity assay. GPx activity in tissue homogenates was measured by the assay described by Sun *et al.* [16]. Catalase activity was determined by measuring the decomposition of hydrogen peroxide at 520 nm using the Catalase-520TM assay kit (OxisResearchTM, Portland, OR). MnSOD and Cu/ZnSOD levels were measured by activity gels as previously described [17,18]. Images of the gels were analyzed by ImageQuant software.

### Reactive oxygen species (ROS) production from mitochondria

H2O2 release from isolated mitochondria obtained from skeletal muscle was measured using the fluorogenic probe, Amplex Red (Molecular Probes, Eugene, OR) as previously described [19].

### Assays for lipid peroxidation (F2-isoprostane levels)

The levels of F2-isoprostanes were determined using gas chromatography/mass spectrometry as described by Morrow and Roberts [20]. The plasma samples obtained from young (4-6 months old) Tg(TXN2)^+/0^ and WT mice were added to HPLC (pH 3.0) water and mixed by vortex. After centrifugation (2,500 x g for 3 minutes at 4°C), the F2-isoprostanes were extracted from the clear supernatants with a C18 Sep-Pak column and a silica Sep-Pak column. The F2-isoprostanes were then converted to pentafluorobenzyl esters and subjected to thin layer chromatography. The F2-isoprostanes were further converted to trimethylsilyl ether derivatives, and the F2-isoprostane levels were quantified by gas chromatography/mass spectrometry. An internal standard, 8-isoPGF2a-d4 (Cayman Chemical, Ann Arbor, MI), was added to the samples at the beginning of extraction to correct the yield of the extraction process. The amounts of F2-isoprostanes were expressed as picograms of 8-Iso-prostaglandin F2 per milliliter of plasma sample.

### Assays for DNA oxidation (8-oxodG levels)

The levels of oxidative damage to DNA were measured by the amount of 8-oxo-2-deoxyguanosine (oxo8dG) in DNA as described by Hamilton *et al.* [21]. DNA was isolated from liver obtained from young (4-6 months old) Tg(TXN2)^+/0^ and WT mice by NaI extraction using the DNA Extractor WB Kit (Wako Chemicals USA, Inc., Richmond, VA). The data are expressed as the ratio of nmoles of oxo8dG to 105 nmoles of 2dG.

### Survival study

Mice in the survival groups were allowed to live out their lives, and the lifespan for individual mice was determined by recording the age of spontaneous death. A survival study consisting of 19 Tg(TXN2)^+/0^ and 22 WT male mice was conducted. The survival curves were compared statistically using the log-rank test [22]. The mean, median, and 10th percentile (when 90% of the mice had died) survival were calculated for each group. The mean survivals for each experimental group were compared to the respective WT group by performing a Student’s t-test upon log-transformed survival times. The median and 10th percentile survivals for each group were compared to the WT group using a score test adapted from Wang *et al.* [23].

### Cross–sectional pathological assessment

The cross-sectional pathological analyses were conducted with 23 Tg(TXN2)^+/0^ mice and 19 WT male mice. After the gross pathological examinations, the following organs and tissues were excised and preserved in 10% buffered formalin: brain, pituitary gland, heart, lung, trachea, thymus, aorta, esophagus, stomach, small intestine, colon, liver, pancreas, spleen, kidneys, urinary bladder, reproductive system (prostate, testes, epididymis, and seminal vesicles), thyroid gland, adrenal glands, parathyroid glands, psoas muscle, knee joint, sternum, and vertebrae. Any other tissues with gross lesions were also excised. The fixed tissues were processed conventionally, embedded in paraffin, sectioned at 5 m, and stained with hematoxylineosin. The diagnosis of each histopathological change was made with histological classifications in aging mice as previously described [24,25]. A list of pathological lesions was constructed for each mouse that included both neoplastic and non-neoplastic diseases. Based on these histopathological data, the tumor burden, disease burden, and severity of each lesion in each mouse were assessed as previously described [25–27].

### Determination of c-Jun and c-Fos levels

Total cell lysates from liver of young (4-6 months old) Tg(TXN2)^+/0^ and WT mice were prepared, and detection of c-Jun (Catalog No. 9165; Cell Signaling Technology, Inc., Danvers, MA) and c-Fos (Catalog No. 4384; Cell Signaling Technology, Inc., Danvers, MA) were performed using Western blots. The amount of c-Jun and c-Fos was quantified by a densitometer, and the data were expressed as the relative amount of protein in lysates using β-actin as an internal standard.

### Determination of mTOR signaling pathway activity and HIF-1α levels

Levels of p70S6K1 and 4E-BP1 (phosphorylated and non-phosphorylated forms) were measured in the total cell lysates from liver of young (4-6 months old) Tg(TXN2)^+/0^ and WT mice by Western blot analysis using mouse p70S6K1, phospho-p70S6K1, 4E-BP1, and phospho-4EBP1 antibodies (Cell Signaling Technology, Inc., Danvers, MA).

Total cell lysates from liver of young (4-6 months old)Tg(TXN2)^+/0^ and WT mice were prepared, and detection of p70S6K1 and 4E-BP1 (phosphorylated and non-phosphorylated forms) was performed using Western blots. The amount of p70S6K1 and 4E-BP1 (phosphorylated and non-phosphorylated forms) was quantified by a densitometer, and the data were expressed as the relative amount of protein in lysates using β-actin as an internal standard.

Total cell lysates from liver of young (4-6 months old) Tg(TXN2)^+/0^ and WT mice were prepared, and detection of HIF-1α (Catalog No. Ab82832; Abcam, Cambridge, MA) was performed using Western blots. The amount of HIF-1α was quantified by a densitometer, and the data were expressed as the relative amount of protein in lysates using β-actin as an internal standard.

### Measurement of the NFκB pathway

The levels of NFκB (p65 and p50) were measured in the total cell lysates from liver of young (4-6 months old) Tg(TXN2)^+/0^ and WT mice by Western blot analysis using mouse NFκB p65 (Catalog No. 3034; Cell Signaling Technology, Inc., Danvers, MA) and p50 (Catalog No. 3035; Cell Signaling Technology, Inc., Danvers, MA) antibodies.

### Statistical analysis

Unless otherwise specified, all experiments were done at least in triplicate. Data were expressed as means ± SEM and were analyzed by the non-parametric test ANOVA. All pair-wise contrasts were computed using Tukey error protection at 95% CI, unless otherwise indicated. Differences were considered statistically significant at p<0.05.

## Results

### Overexpression of Trx2 in tissues from Tg(TXN2)^+/0^ mice

**Figure 2.**
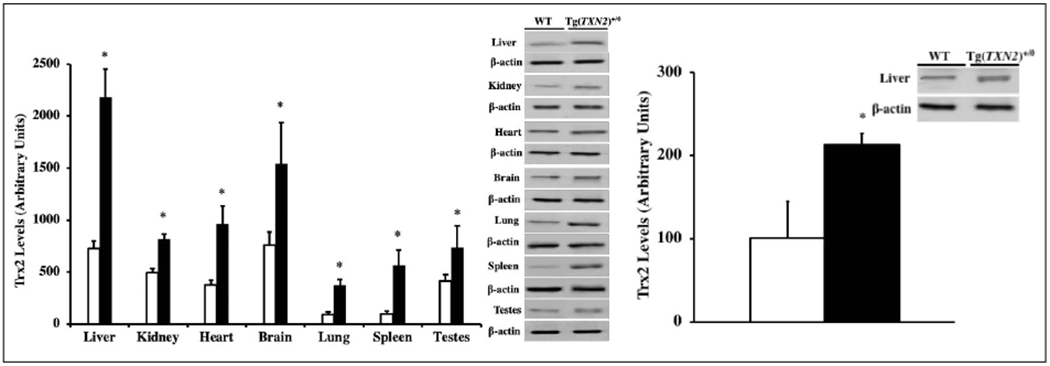
Overexpression of Trx2 in young and old Tg(TXN2)^+/0^ mice and their WT littermates. The levels of Trx2 protein were determined by Western blot in various tissues of 4-6 months old (Figure 2a: left) and in the liver of 22-24 months old (Figure 2b: right) Tg(TXN2)^+/0^ (closed bar) and WT (open bar) mice. Trx2 levels were significantly higher in both young and old Tg(TXN2)^+/0^ mice compared to their WT littermates (*p< 0.05). The data are the mean SEM from three to five mice.

The levels of Trx2 in tissues (liver, kidney, heart, brain, lung, spleen, and testes) from young (4-6 months old) Tg(TXN2)^+/0^ and WT mice were measured using Western blot analysis. The Trx2 protein levels were significantly higher (approximately 1.6- to 5-fold) in all of the seven tissues examined in young Tg(TXN2)^+/0^ mice compared to their WT littermates (Figure 2a; p< 0.05). The levels of Trx2 overexpression were maintained in the liver of old (22-24 months old) Tg(TXN2)^+/0^ mice (Figure 2b; p< 0.05).

### Levels of Trx1, glutaredoxin, total glutathione, and major antioxidant enzymes in tissues from Tg(TXN2)^+/0^ mice

**Figure 3.**
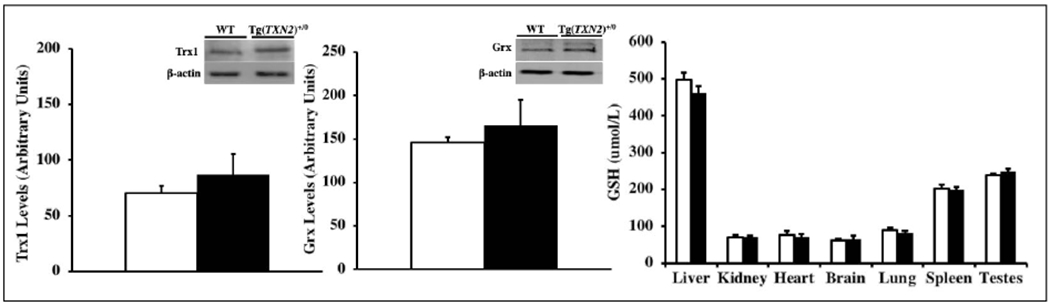
Levels of Trx1, glutaredoxin, and total glutathione in Tg(TXN2)^+/0^ and WT mice. The levels of Trx1 (Figure 3a: left), glutaredoxin (Figure 3b: center), and total glutathione (Figure 3c: right) were measured in the liver of 4-6 months old Tg(TXN2)^+/0^ (closed bar) and WT mice (open bar). No significant difference was observed in Trx1, glutaredoxin, or total glutathione in Tg(TXN2)^+/0^ mice compared to WT mice. The data in figures 3a–3c are the mean SEM from three to five mice.

To examine whether the increased levels of Trx2 altered the levels of Trx1, glutaredoxin, and glutathione, which have biological functions similar to Trx2, we measured the levels of Trx1, glutaredoxin, and total glutathione in liver and other tissues obtained from young (4-6 months old) Tg(TXN2)^+/0^ and WT mice. The data in Figure 3 show that Trx1 (Figure 3a) and glutaredoxin (Figure 3b) levels in the liver were similar between Tg(TXN2)^+/0^ and WT mice at 4-6 months of age (p> 0.05). The levels of total glutathione in the tissues (liver, kidney, heart, brain, lung, spleen, and testes) were also similar between young (4-6 months old) Tg(TXN2)^+/0^ and WT mice (Figure 3c; p> 0.05).

The activities of major antioxidant enzymes (Cu/ZnSOD, MnSOD, glutathione peroxidase (GPx), and catalase) were similar between young (4-6 months old) Tg(TXN2)^+/0^ and WT mice (data not shown).

### Hydrogen peroxide production from mitochondria

**Figure 4.**
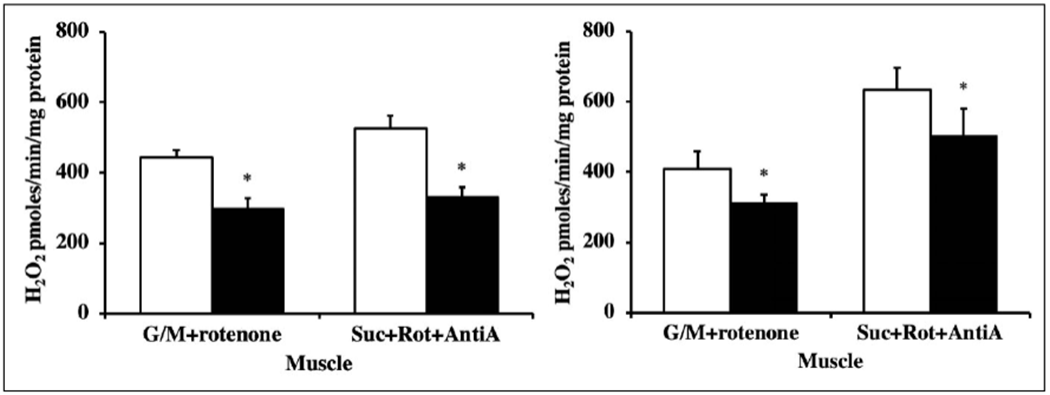
Hydrogen peroxide production in young and old Tg(TXN2)^+/0^ and WT mice. The Amplex Red assay was performed in the skeletal muscle of 4-6 months (Figure 4a: left) and 22-24 months old (Figure 4b: right) Tg(TXN2)^+/0^ (closed bar) and WT (open bar) mice. Under different experimental conditions, H2O2 production was significantly less in mitochondria from both young and old Tg(TXN2)^+/0^ mice compared to WT mice (*p< 0.05). The values are the mean ± SEM of five mice per group.

H_2_O_2_ release from isolated mitochondria obtained from skeletal muscle was measured using the fluorogenic probe, Amplex Red (Molecular Probes, Eugene, OR) as previously described [19]. The H_2_O_2_ release from isolated mitochondria from skeletal muscle was significantly less in both young (4-6 months old) and old (22-24 months old) Tg(TXN2)^+/0^ mice compared to their WT littermates (Figures 4a and 4b; p< 0.05).

### Lipid peroxidation (F2-isoprostane levels) and DNA oxidation (8-oxodG levels)

**Figure 5.**
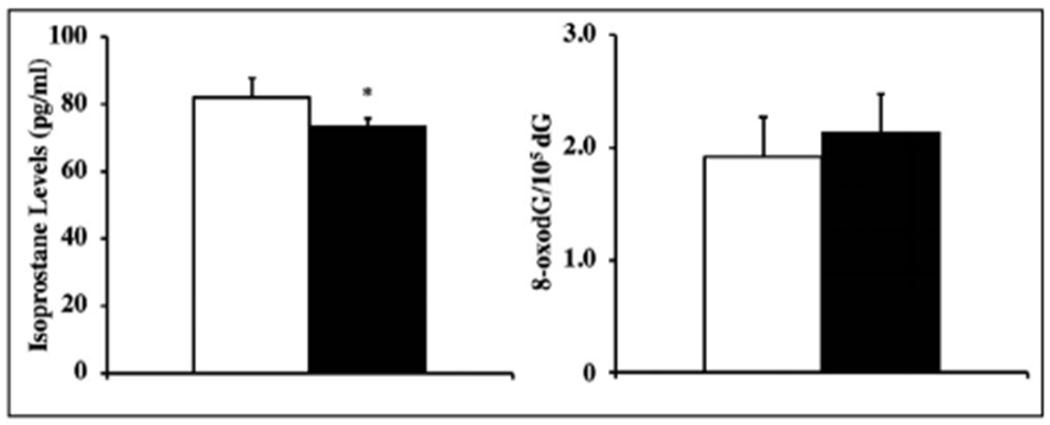
Levels of F2-isoprostanes and DNA oxidation in Tg(TXN2)^+/0^ and WT mice. F2-isoprostanes levels were measured in plasma samples from 4-6 months old (Figure 5a: left) and DNA oxidation (8-oxodG) in liver from 4-6 months old (Figure 5b: right) Tg(TXN2)^+/0^ (closed bar) and WT (open bar) mice. The F2-isoprostane levels were significantly lower in Tg(TXN2)^+/0^ mice than in WT control mice (*p< 0.05). Levels of DNA oxidation in the livers of Tg(TXN2)^+/0^ and WT mice showed no significant difference. The data are the mean SEM from five mice.

We tested whether Trx2 overexpression protects against oxidative damage by measuring levels of lipid peroxidation (F2 isoprostanes) in serum and DNA oxidation (8-oxodG) in liver from young (4-6 months old) Tg(TXN2)^+/0^ and WT mice. Levels of F2-isoprostanes were significantly lower in young (4-6 months old) Tg(TXN2)^+/0^ mice compared to WT mice (Figure 5a; p< 0.05), however, Tg(TXN2)^+/0^ mice had only 13-14% lower levels of F2-isoprostanes compared to WT mice, in spite of the significant ROS production from mitochondria (Figures 4a and 4b). Although Trx2 overexpression reduced the ROS production from mitochondria and oxidative damage to lipids, levels of DNA oxidation measured by 8-oxodG were similar between young (4-6 months old) Tg(TXN2)^+/0^ and WT mice (Figure 5b; p> 0.05).

### Survival curves, body and organ weights

**Figure 6.**
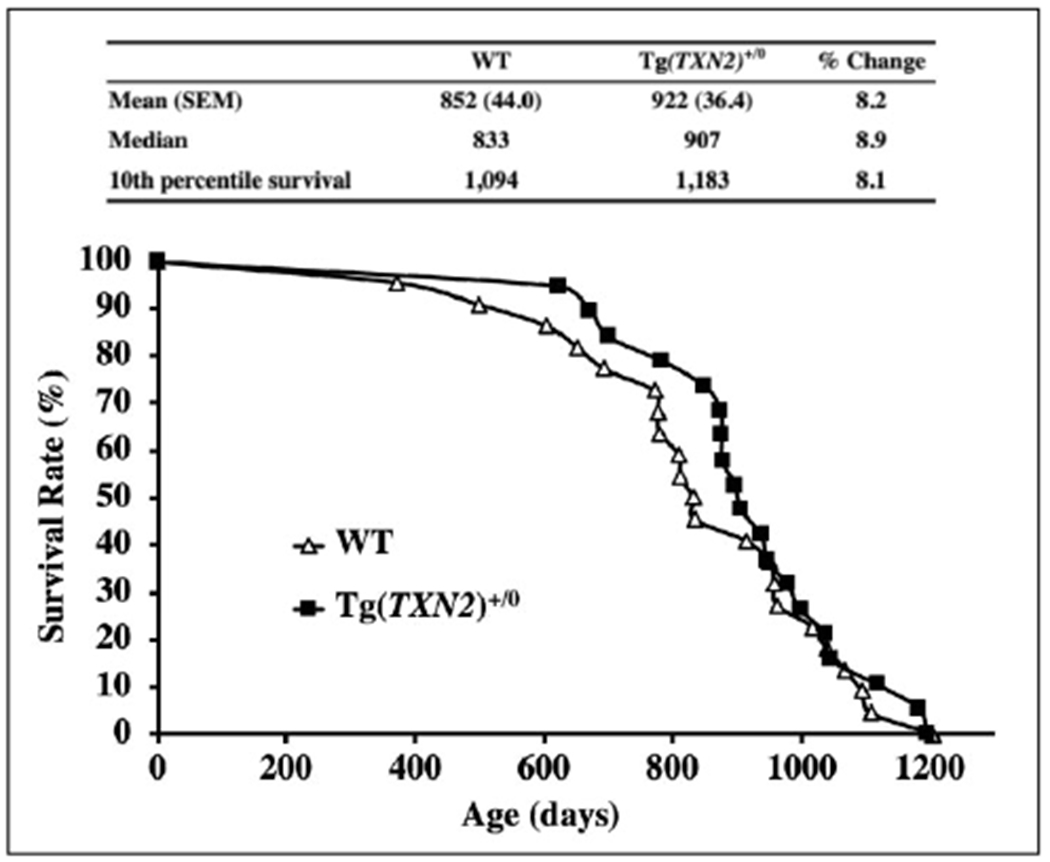
The survival curves of Tg(TXN2)^+/0^ and WT mice. The survival curves, mean, median, and 10th percentile lifespans (days), and percent differences of Tg(TXN2)^+/0^ (closed squares) and WT (open triangles) mice are presented. The cohort consists of 19 Tg(TXN2)^+/0^ and 22 WT male mice. The survival curves did not show a significant difference between Tg(TXN2)^+/0^ and WT mice (p> 0.05). Tg(TXN2)^+/0^ mice had a slightly longer mean (8.2%), median (8.9%), and 10th percentile (8.1%) lifespans compared to WT mice, which were not statistically significant (p> 0.05).

The survival curves of Tg(TXN2)^+/0^ and WT mice are presented in Figure 6. The survival study was conducted with 19 Tg(TXN2)^+/0^ and 22 WT male mice. The survival curves were not significantly different between Tg(TXN2)^+/0^ and WT mice (Log-rank: p> 0.05). The mean, median, and 10th percentile survival for WT mice were 852, 833, and 1,094 days, respectively (Figure 6). The mean, median, and 10th percentile survival for Tg(TXN2)^+/0^ mice were 922, 907, and 1,183 days, respectively (Figure 6). Tg(TXN2)^+/0^ mice had slightly longer mean (8.2%), median (8.9%), and 10th percentile (8.1%) lifespans compared to WT mice, however, these differences did not reach statistical significance (p> 0.05).

The body and organ weights were similar between Tg(TXN2)^+/0^ and WT mice (Table 1). Food consumption was also similar between Tg(TXN2)^+/0^ and WT mice (data not shown).

### Cross-sectional pathology

The cross-sectional pathological analyses of 23 Tg(TXN)^+/0^ and 19 WT mice (22-24 months old) showed that the major disease in these mice was neoplastic disease. The tumors observed were lymphoma, hemangioma/hemangiosarcoma in the liver and spleen, pulmonary adenocarcinoma, hepatocellular carcinoma, and adenoma in the thyroid gland, and the most prevalent tumor was lymphoma, which is consistent with the pathology data from C57BL/6 mice and mice overexpressing Trx1 [11,13].

**Figure 7.**
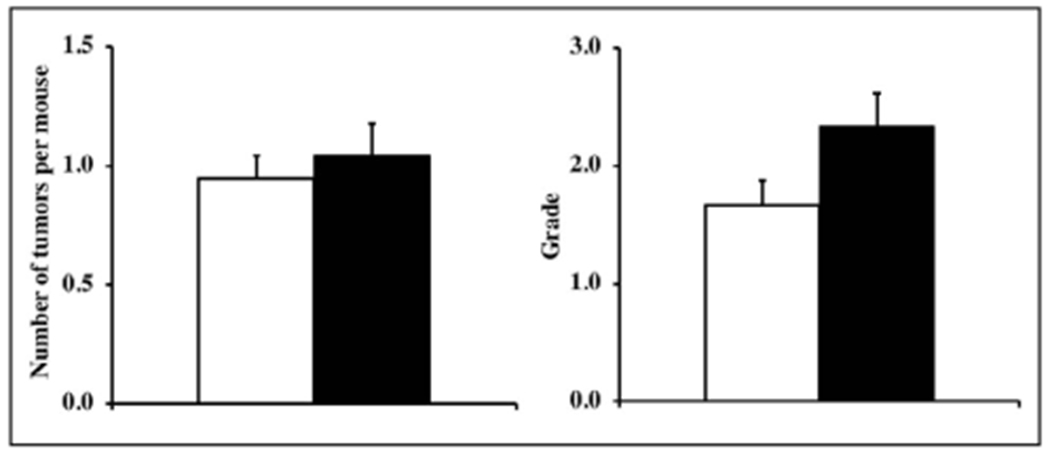
Tumor burden and severity of lymphoma in Tg(TXN2)^+/0^ and WT mice. The number of different types of tumors, tumor burden (Figure 7a: left) and the severity of lymphoma (Figure 7b: right) in Tg(TXN2)^+/0^ (closed bar) and WT (open bar) mice were compared at 22-24 months old. The cohort consists of 23 Tg(TXN2)^+/0^ and 19 WT male mice. The tumor burden for the Tg(TXN2)^+/0^ mice is similar to WT mice and the severity of lymphoma is slightly higher in Tg(TXN2)^+/0^ mice compared to their WT littermates, which were not statistically significant (p> 0.05).

First, we compared the number of different types of tumors (tumor burden) for each mouse in both Tg(TXN2)^+/0^ and WT groups because aging mice may have several different types of tumors. As the data in Figure 7a show, the tumor burden for the Tg(TXN2)^+/0^ mice is similar to WT mice (p > 0.05). The incidence of lymphoma was similar between Tg(TXN2)^+/0^ and WT mice (data not shown). Tg(TXN2)^+/0^ mice had a slightly higher severity of lymphoma than WT mice, which was not statistically significant (Figure 7b; p> 0.05).

Next, we compared the severity of major non-neoplastic disease between the two groups. Severity of glomerulonephritis and inflammation, which were the most common non-neoplastic lesions observed in these mice, were compared between Tg(TXN2)^+/0^ and WT mice. No significant changes were found regarding the severity of glomerulonephritis or inflammation in Tg(TXN2)^+/0^ and WT mice (data not shown). The disease burden, defined as the total number of histopathological changes in a body, which can also serve as a good index of age-related accumulation of tissue and cell injury [24,26,27], were similar between Tg(TXN2)^+/0^ and WT mice (data not shown).

### Measurement of the c-Fos and c-Jun levels

**Figure 8.**
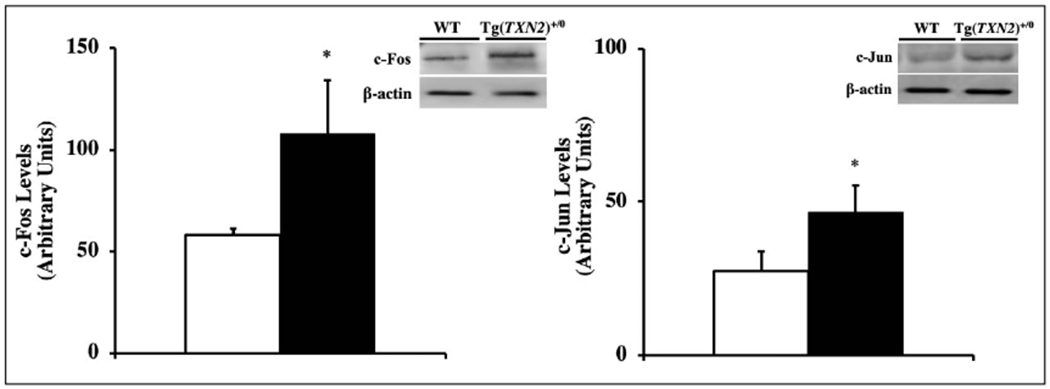
Levels of c-Fos and c-Jun in Tg(TXN2)^+/0^ and WT mice. The levels of c-Fos (Figure 8a: left) and c-Jun (Figure 8b: right) were measured in the liver of 4-6 months old Tg(TXN2)^+/0^ (closed bar) and WT mice (open bar) by Western blot. The c-Fos and c-Jun levels were significantly higher in Tg(TXN2)^+/0^ mice than in WT control mice (*p< 0.05). The data are the mean SEM from three to five mice.

Since c-Fos and c-Jun are redox-sensitive transcription factors, and their levels are correlated to cancer development, the levels of c-Fos and c-Jun were measured using Western blot. The data in Figure 8 show that Tg(TXN2)^+/0^ mice had significantly higher levels of c-Fos and c-Jun compared to their WT littermates (Figures 8a and 8b, respectively; p< 0.05).

### Determination of mTOR signaling pathway activity and HIF-1α levels

**Figure 9.**
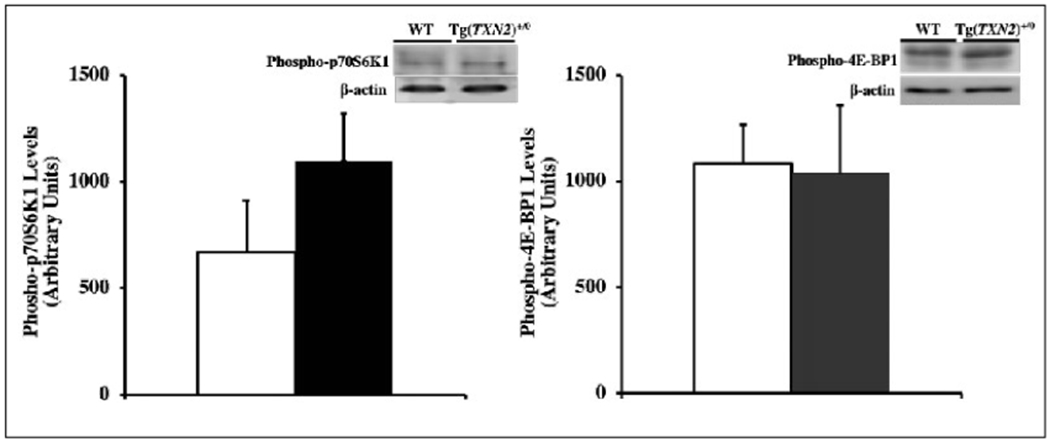
Levels of mTOR in Tg(TXN2)^+/0^ and WT mice. The levels of phospho-p70S6K1 (Figure 9a: left) and phospho-4E-BP1 (Figure 9b: right) were measured in the liver of young (4-6 months old) Tg(TXN2)^+/0^ (closed bar) and WT (open bar) mice by Western blot analysis. The levels of phospho-p70S6K1 and phospho-4E-BP1 were similar between Tg(TXN2)^+/0^ and WT mice (p> 0.05). The data are the mean SEM from three to five mice.

Since substantial evidence showed that mTOR activity is one of the key pathways for cancer development and lifespan, levels of p70S6K1 and 4E-BP1 (phosphorylated and non-phosphorylated forms) were measured in the total cell lysates from liver of Tg(TXN2)^+/0^ and WT mice by Western blot analysis using mouse p70S6K1, phospho-p70S6K1, 4E-BP1, and phospho-4E-BP1 antibodies. The data in Figure 9a show that Tg(TXN2)^+/0^ mice had slightly higher levels of phospho-p70S6K1 compared to WT littermates, although the difference was not statistically significant (Figure 9a; p> 0.05). Levels of phospho-4E-BP1 were similar between Tg(TXN2)^+/0^ and WT mice (Figure 9b; p> 0.05). In addition, the levels of HIF-1α, which also plays important roles in cancer development, were similar between Tg(TXN2)^+/0^ and WT littermates (data not shown).

### Measurement of the NFκB pathway

**Figure 10.**
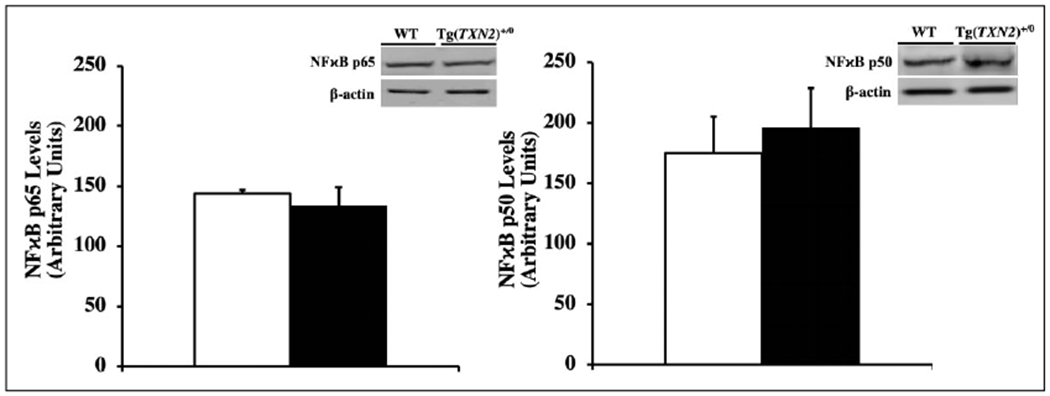
Levels of NFκB p65 and NFκB p50 in Tg(TXN2)^+/0^ and WT mice. The levels of NFκB p65 (Figure 10a: left) and NFκB p50 (Figure 10b: right) were measured in the liver of young (4-6 months old) Tg(TXN2)^+/0^ (closed bar) and WT (open bar) mice. The levels of NFκB p65 and NFκB p50 were similar between Tg(TXN2)^+/0^ and WT mice (p> 0.05). The data are the mean SEM from three to five mice.

Since NFκB is one of the redox-sensitive transcription factors, which plays important roles in oxidative stress, inflammation, apoptosis, and cancer, the levels of NFκB (p65 and p50) were measured using Western blot. The data in Figure 10 show that Tg(TXN2)^+/0^ mice had similar levels of NFκB p65 and NFκB p50 compared to their WT littermates (Figures 10a and 10b, respectively; p> 0.05).

## Discussion

After the initial discovery as the major reductant for a variety of enzymes in the early 1960s, unique biological roles of thioredoxin (Trx) have been well documented [1]. Trx plays important roles as a hydrogen donor to enzymes involved in reductive reactions [e.g., ribonucleotide reductase, which reduces ribonucleotides to deoxyribonucleotides for DNA synthesis; peroxiredoxin (Prx), which reduces peroxides [4–6]and methionine sulfoxide (MetO) reductase, which reduces MetO in proteins and provides protection against oxidative stress [2,3,7]. Trx also plays important roles to protect cells and tissues from oxidative stress by maintaining a reduced environment in cells through thiol-disulfide exchange reactions [10,28–30]. Trx has two forms in human cells: one in cytosolic (hTrx1) [8] and another in mitochondrial (hTrx2) [9]. Essential roles of Trx1 and Trx2 in mammals were further proven by the studies with knockout mice null for either Trx1 or Trx2, which are embryonically lethal [31,32]. Although the important roles of Trx along with thioredoxin interacting protein (Txnip) in pathophysiology have been well documented [1,33], the exact roles of Trx on aging and age-related pathology in mammals had been largely unknown.

To test the role of Trx in aging, a lifespan study was first conducted using the mice overexpressing Trx1 generated with a transgene containing the human thioredoxin cDNA fused to the β-actin promoter [Tg(act-TXN)^+/0^ mice] [10,12]. The study demonstrated that Trx1 overexpression resulted in an increased lifespan compared to their WT littermates [10,12]. However, there were shortcomings in the study; the survival study was conducted under conventional housing conditions and the lifespan of WT C57BL/6 mice in their colony was shorter than WT C57BL/6 mice in aging colonies under optimal conditions. Thus, our laboratory conducted an aging study to examine the effects of increased levels of Trx1 on oxidative stress and aging under optimal housing conditions [13], using Tg(act-TXN)^+/0^ mice. Our study demonstrated that Trx1 overexpression showed a significant increase in the survival of male Tg(act-TXN)^+/0^ mice compared to WT mice only during the first half of their lifespan, but no increase of lifespan was observed in later part of life. Tg(act-TXN)^+/0^ mice showed that the levels of overexpression significantly decreased with age possibly due to the β-actin promoter driving expression of the transgene [13]. Subsequently, we generated new transgenic mice with clones of the human TXN gene containing endogenous promoters [Tg(TXN)^+/0^] to test the effects of continuous Trx1 overexpression over the lifespan on aging and age-related diseases [11]. Tg(TXN)^+/0^ mice showed some beneficial effects in the earlier part of life but had no significant effects on median or maximum lifespans, which was accompanied with accelerated cancer development in old mice [11]. These results are consistent with the observations in Tg(act-TXN)^+/0^ mice.

The results from two lines of Trx1 transgenic mice clearly demonstrated that overexpression of Trx1 in cytosol has beneficial effects only in the earlier part of life. These results also led us to question whether increased expression of Trx2 in the mitochondria plays more important roles in aging because the importance of antioxidant overexpression in mitochondria in aging and age-related disease was strongly suggested by a study using mice overexpressing catalase in mitochondria [14]. In their study, overexpressing catalase in the mitochondria significantly extended lifespan and attenuated age-related diseases compared to their WT littermates, while overexpressing catalase in the nucleus or peroxisome did not have beneficial effects on aging [14]. Thus, the purpose of this study was to test the effects of Trx2 overexpression in the mitochondria on aging and age-related diseases using male Tg(TXN2)^+/0^ C57BL/6 mice.

To directly test the effects of Trx2 overexpression on aging and age-related diseases, we generated the Tg(TXN2)^+/0^ mice using the human thioredoxin 2 gene. Young (4-6 months old) male Tg(TXN2)^+/0^ mice showed that levels of Trx2 were significantly higher in all the tissues examined compared to WT control mice. The levels of Trx2 were 1.6- to 5-fold higher in all of the tissues examined. The increased levels of Trx2 in male Tg(TXN2)^+/0^ mice did not alter Trx1, glutaredoxin or total glutathione levels. The activities of major antioxidant enzymes (Cu/ZnSOD, MnSOD, glutathione peroxidase (GPx), and catalase) were also similar between young (4-6 months old) Tg(TXN2)^+/0^ and WT mice. The Trx2 overexpression suppressed the hydrogen peroxide production from isolated skeletal muscle mitochondria from both young (4-6 months old) and old (22-24 months old) Tg(TXN2)^+/0^ mice compared to WT control mice as expected.

Although the skeletal muscle mitochondria obtained from Tg(TXN2)^+/0^ mice showed less hydrogen peroxide production compared to WT control mice, the effects of Trx2 overexpression on oxidative damage were minimal. Plasma isoprostane levels were approximately 13-14% less in Tg(TXN2)^+/0^ compared to WT control mice, however, the levels of DNA oxidation measured by 8-oxodG were similar between Tg(TXN2)^+/0^ and WT control mice.

Our survival study showed that the survival curve of male Tg(TXN2)^+/0^ mice was not significantly different from WT control mice. Although the mean, median, and 10th percentile lifespans of male Tg(TXN2)^+/0^ mice were approximately 8-9% longer than WT control mice, these differences were not statistically significant. The cross-sectional pathology showed that the total number of tumors (tumor burden) was similar between Tg(TXN2)^+/0^ (1.04) and WT control mice (0.95). The incidence of lymphoma, a major neoplastic disease, was also similar between Tg(TXN2)^+/0^ and WT control mice. The severity of lymphoma was slightly higher in Tg(TXN2)^+/0^ mice compared to WT mice, although the difference was not statistically significant. These pathological observations indicate that overexpression of Trx2 in mitochondria may play a similar role in the development and growth of lymphoma as the overexpression of Trx1 [11,13]. i.e., accelerate the growth and development of lymphoma as they age.

Subsequently, we measured several signaling pathways (i.e., mTOR, NFκB, and c-Jun/Fos) because substantial evidence shows that these pathways play important roles in cancer development and lifespan and can also be attenuated by Trx. Although overexpression of Trx2 in mitochondria suppressed the ROS production from mitochondria and reduced levels of lipid peroxidation, its effects on signaling pathways were minimal. Our data showed the levels of phospho-p70S6K1, phospho-4E-BP1, NFκB p65, and NFκB p50 were similar between Tg(TXN2)^+/0^ and WT littermates, which suggests that the mTOR and NFκB pathways were not changed by the overexpression of Trx2 in mitochondria. On the other hand, we saw increased levels of c-Jun and c-Fos in Tg(TXN2)^+/0^ mice compared to their WT littermates. Activator protein 1 (AP-1), is a complex of proteins of the Jun and Fos families. In mammals, three Jun proteins (c-Jun, Jun B, and Jun D) and four Fos family proteins (c-Fos, Fos B, Fra-1, and Fra-2) have been identified. AP-1 proteins bind to TPA-response elements (TRE) or AP-1 binding sites to transcriptionally activate effector genes, which have been shown to stimulate cell proliferation and transformation. AP-1 DNA binding activity has been shown to be enhanced by Trx via Ref-1 by the reduction of a single conserved cysteine residue in the DNA binding domain of each subunit [34]. Therefore, increased levels of c-Fos and c-Jun may contribute to slightly accelerated cancer development in Tg(TXN2)^+/0^ mice.

Although the minimal effects of Trx2 overexpression on aging and age-related pathology are somewhat disappointing, the outcome of this study along with our previous works with the mice overexpressing Trx1 could indicate overexpression of Trx in either cytosol or mitochondria alone may have limited biological effects. In other words, synergetic effects of Trx1 and Trx2 may be required to have more robust effects on pathophysiology during aging. This notion is supported by our recent study that overexpression of Trx in both the cytosol and mitochondria in TXNTg x TXN2Tg mice unexpectedly had a shorter lifespan and enhanced tumor development compared to WT mice [35]. To further test the synergetic effects of Trx overexpression in the cytosol and mitochondria on aging and age-related disease, our laboratory is currently testing if the down-regulation of Trx in both the cytosol and mitochondria could have anti-aging and/or anti-cancer effects.

## Figures and Tables

**Table 1.** Body and organ weights of Tg(TXN2)^+/0^ young mice.

	WT(n=6)	Tg(TXN2)^+/0^(n=6)
Body Weight (g)	30.711± 1.222	29.198 ± 2.138
Liver (g)	1.847 ± 0.146	1.688 ± 0.126
Spleen (g)	0.100 ± 0.007	0.132 ± 0.026
Pancreas (g)	0.185 ± 0.012	0.185 ± 0.024
Heart (g)	0.177 ± 0.006	0.187 ± 0.015
Lung(g)	0.207 ± 0.006	0.197 ± 0.010
Left Kidney (g)	0.272 ± 0.009	0.256 ± 0.031
Right Kidney (g)	0.302 ± 0.018	0.301 ± 0.020
Left Testis (g)	0.115 ± 0.004	0.120 ± 0.005
Right Testis (g)	0.118 ± 0.005	0.126 ± 0.005
Brain (g)	0.408 ± 0.012	0.412 ± 0.008
